# Collargol in Various Affections

**Published:** 1907-07

**Authors:** 


### Collargol in Various Affections.

In a paper read before the San Angelo District Medical Association (*Texas State Journal of Medicine,* March, 1907), Dr. I. L. Von Zandt of Fort Worth reviews some of the publications on collargol and records his own experience with it in septicemia, scarlatina, phlebitis, erysipelas, bubo, paronychia, acute salpingitis and exacerbations. He also gave it in 13 cases of typhoid fever, with an average of 19.8 days from the first to the last visit. This includes one with intercurrent pneumonia and two who suffered a set back from excitement and overeating. In two cases the temperature had fallen to 99°; when the remedy was discontinued, the temperature remained stationary for a few days and then again rose till collargol was resumed, whereupon fever disappeared in three days. In most cases there was marked improvement with general well-being in two or three days after beginning the treatment, the fever some times coming to a very abrupt termination.

